# Supplementary material for: Microwave resonances of magnetic skyrmions in thin film multilayers
Source: Nat Commun. 2021 Mar 26;12:1909. doi: 10.1038/s41467-021-22220-1 (PMC7998029; doi:10.1038/s41467-021-22220-1)
Supplement: Supplementary file 1 — Supplementary Information [file 41467_2021_22220_MOESM1_ESM.pdf]

Supplementary Materials — Microwave Resonances of Magnetic Skyrmions in Thin Film Multilayers

Bhartendu Satywali[1,⋆], Volodymyr P. Kravchuk[2,3,⋆],
Liqing Pan[4], M. Raju[1], Shikun He[1], Fusheng Ma[1],
A.P. Petrović[1,†], Markus Garst[3,5,6], Christos Panagopoulos[1,†]

[1]*Division of Physics and Applied Physics,*
School of Physical and Mathematical Sciences,
Nanyang Technological University, 637371 Singapore.

[2]*Bogolyubov Institute for Theoretical Physics of National*
Academy of Sciences of Ukraine, 03680 Kyiv, Ukraine.

[3]*Institute for Theoretical Solid State Physics,*
Karlsruhe Institute of Technology, 76131 Karlsruhe, Germany.

[4]*Research Institute for Magnetoelectronics and Weak Magnetic Field Detection,*
College of Science, China Three Gorges University, 443002 Yichang, China.

[5]*Institut für Theoretische Physik, TU Dresden, 01062 Dresden, Germany.*

[6]*Institute for Quantum Materials and Technologies,*
Karlsruhe Institute of Technology, 76021 Karlsruhe, Germany.

⋆ *Equal Contribution,* † *Corresponding Authors*
appetrovic@ntu.edu.sg, christos@ntu.edu.sg

CONTENTS

I. Experiments 3

A. Magnetic characterisation 3

B. Fitting of absorption spectra in the field-polarised and skyrmion phases 5

C. Temperature dependence of resonance spectra 12

II. Micromagnetic simulations 13

A. Magnetic parameters for mumax^3 simulations 13

B. Determination of exchange stiffness A and Dzyaloshinskii-Moriya interaction D 13

C. Influence of inter-layer dipolar coupling on the resonance spectra 15

D. Annealing the metastable disordered skyrmion lattice configuration 16

E. Micromagnetic simulations of a metastable state with a single skyrmion in a field-polarised background 18

F. Micromagnetic simulations of a unit cell of a hexagonal skyrmion lattice 19

1. Size of the unit cell and averaged topological density 19

2. Relation between frequency of the LF mode and skyrmion radius R_{sk} 19

3. Breathing mode in $[Ir_1Fe_{0.5}Co_{0.5}Pt_1]^{20}$ multilayers 21

III. Analytical theory of a single skyrmion in multilayers 21

A. Model 22

B. Static skyrmion solution 23

C. Spin wave excitation in the presence of a skyrmion 25

IV. Supplementary videos 27

A. LF oscillations for configuration 1: a single skyrmion in a field-polarised background 27

B. HF, LF and breathing (BR) oscillations for configuration 2: a unit cell of a hexagonal skyrmion lattice 27

References 28

I. EXPERIMENTS

A. Magnetic characterisation

Multilayers of $[\mathrm{Ir_1Fe_{0.5}Co_{0.5}Pt_1}]^{20}$ were grown at room temperature on thermally-oxidised Si wafers using dc magnetron sputtering. The details of the deposition procedure are included in references [1–3]. Magnetometry measurements were performed in a Quantum Design™ MPMS-XL SQUID magnetometer. Figure S1a depicts two magnetisation $M(H)$ hysteresis loops acquired at 100 K and 300 K. The saturation magnetisation M_s increases as the temperature is reduced, as expected for the field-polarised state. The saturation fields for in-plane and out-of-plane directions only differ slightly from each other at 300 K but this difference increases drastically reaching ≈ 300 mT as the temperature is reduced to 100 K (Fig S1b). Ferromagnetic resonance (FMR) spectra in the field-polarised state were recorded using the field-sweep method for magnetic fields applied out-of-plane (OP). Figure S1c shows microwave absorption spectra S_{12} at 300 K acquired at intervals of 1 GHz ranging from 15 up to 25 GHz. In the field-polarised state, each experimental spectrum is fitted with an asymmetric Lorentzian model (see the Methods section in the main text for details) to extract its resonance field and linewidth at the measurement frequency. In Fig. S1d the extracted resonances were fitted to the Kittel formula

$$2\pi f = \frac{g\mu_B}{\hbar}\mu_0\left(H_\perp + \frac{2K}{\mu_0 M_s} - M_s\right), \tag{1}$$

where the universal constants μ_B, μ_0 and $\hbar$ are the Bohr magneton, the vacuum permeability, and the reduced Planck constant, respectively; M_s is the saturation magnetisation, K is the uniaxial anisotropy, and g is the g-factor. This formula provides an accurate description of our data when using the saturation magnetisation M_s determined from the magnetisation measurements and fitting the g-factor as well as the uniaxial anisotropy. For 300 K we obtain the following fit parameters: $M_s = 1.02$ MA/m, $K = 0.65$ MJ/m^3 and $g = 2.17$.

We estimate the effective damping α_{eff} in the field-polarised state of our multilayers from a linear fit to the linewidth $\Delta H_\perp$ vs. frequency f (shown in Fig.S1e) using the equation

$$\Delta H_\perp = \frac{2}{\sqrt{3}}\frac{\alpha_{\mathrm{eff}}\hbar}{g\mu_0\mu_B}2\pi f + \Delta H_\perp^0\,. \tag{2}$$

The large extracted $\alpha_{\mathrm{eff}} = 0.05$ at 300 K can be partially attributed to spin-pumping effects at the 40 ferromagnet-heavy metal interfaces in our multilayers. However, the large value of the zero-frequency linewidth $\Delta H_\perp^0 \approx 28$ mT suggests a significant external contribution to $\Delta H_\perp$. This large $\Delta H_\perp^0$ is likely originates from the sputtering growth process of our films which creates

defects and disorder, especially at sub-nanometre thicknesses.

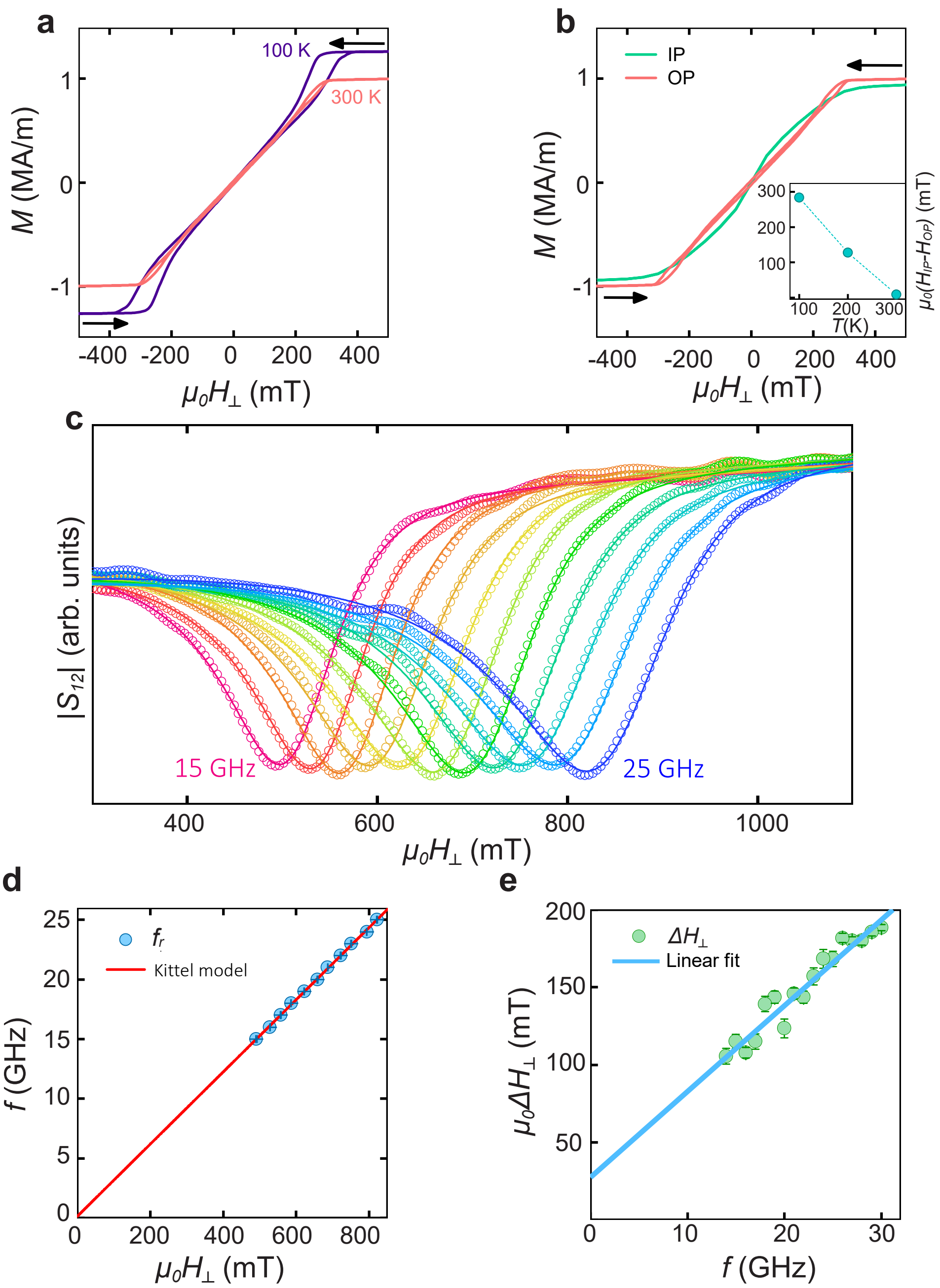

FIG. S1. Ferromagnetic resonance of the saturated field-polarised state in $[Ir_1Fe_{0.5}Co_{0.5}Pt_1]^{20}$. **a** Out-of-plane magnetisation hysteresis loops at 300 K and 100 K. Arrows indicate the field sweep direction. **b** magnetisation hysteresis loops at 300 K with the field applied perpendicular (OP) and parallel (IP) to the film plane. The value for the saturation field differs only slightly for OP and IP applied fields. Inset: The difference in saturation fields rises as the temperature is reduced. **c** Field sweep FMR spectra from 15-25 GHz at 300 K (coloured data-points) with asymmetric peak-fits (lines). **d** Evolution of the ferromagnetic resonance frequency f_r with magnetic field extracted from spectra in (c) and fitted with the Kittel formula Eq. (1). **e** Evolution of the spectral linewidth with resonance frequency, extracted from spectra in (c) and fitted with Eq. (2).

B. Fitting of absorption spectra in the field-polarised and skyrmion phases

Figures S2-S5 show the fits to individual absorption spectra used to extract the datapoints overlaid on Figs. 2a,d. All our experimental spectra have been modelled using standard methods. We have used a single peak Dysonian fit [4] to extract the resonance frequencies and linewidth from our frequency sweep spectra. Similarly, Lorentz functions were used to fit our field sweep data. In this case, a superposition of two Lorentz functions with symmetric and antisymmetric components [5, 6] was used to fit the HF and FM modes simultaneously, for frequencies above 8.4 GHz. Below 8.4 GHz, optimal fits were obtained by using a single Lorentz function. For both frequency and field sweeps, additional constant and linear terms were added to describe the background signal and drift in vector network analyser (VNA) stability over time, respectively [7].

We extract the linewidths $\mu_0 \Delta H_\perp$ for each resonance from our raw field-swept spectra (Fig. S4), and plot them as a function of frequency in Fig. S6a. The gradient $d\mu_0 \Delta H_\perp / df$ is proportional to the damping experienced by each mode. A damping hierarchy clearly develops for our three resonances, with the FM and LF modes experiencing the lowest and highest dampings, respectively.

An enhanced mode-specific damping for the LF resonance is a plausible explanation for our failure to resolve this mode in linecuts parallel to the frequency axis (Figs. 2b,e, main text). To explore this suggestion, we have performed additional simulations of the entire absorption spectrum at higher damping values (Fig. S6b). In our experimentally-accessible field range (6-14 GHz), the simulated LF and HF modes begin to merge, rendering them indistinguishable above $\alpha_{eff} = 0.2$. This observation is consistent with our experimental data. There are a variety of possible causes for enhanced damping in resonances involving non-collinear spin textures. Firstly, the damping is expected to depend on the ellipticity of the modes, resulting in a mode-dependent linewidth [8]. Although this effect is in principle already captured by the micromagnetic simulations, further shape anisotropies of the experimental setup could possibly influence the ellipticity, thus enhancing the damping. However, the magnitude of this effect is expected to be relatively small. Secondly, there are several further sources of damping beyond the Gilbert phenomenology which are not taken into account in the simulations, e.g. magnon scattering of defects, spatial variations of anisotropy energies and the wavevector distribution of the coplanar waveguide (CPW). In particular, the extrinsic damping induced by inhomogeneities could affect the various modes quite differently depending on the extent of the magnon wavefunctions. Inhomogeneities are known to affect the nucleation and position

of individual skyrmions in sputtered multilayers. The LF mode is localised on these individual skyrmions and its properties (including resonance frequency and linewidth) are thus sensitive to the local environment. This might effectively broaden the resonance in the absorption spectra. In contrast, the magnon probabilities of the HF skyrmion mode as well as the FM mode are delocalised and hence effectively averaged over the disorder.

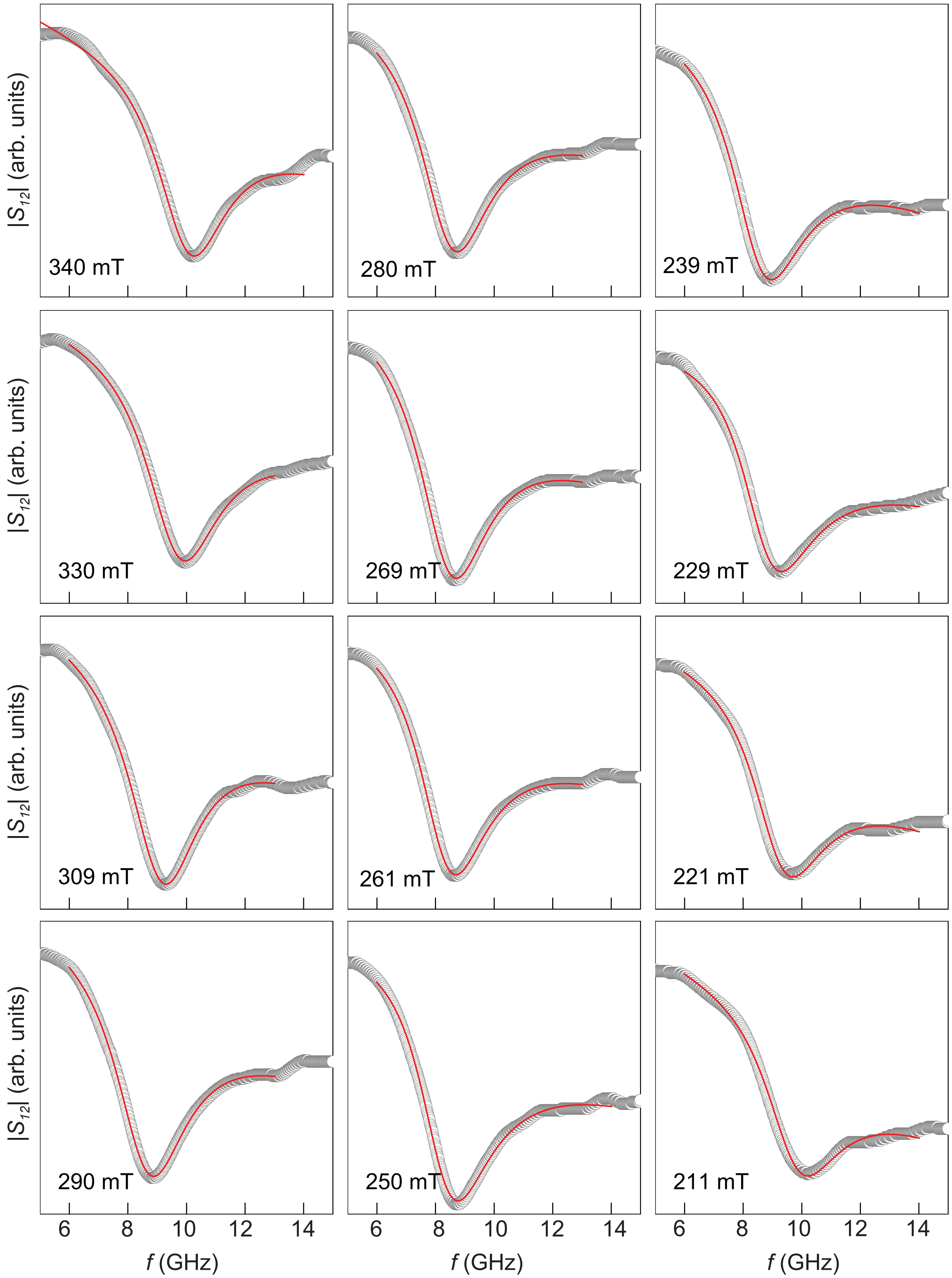

FIG. S2. Experimentally obtained frequency sweep data (grey) fitted with a single peak Dysonian function (red).

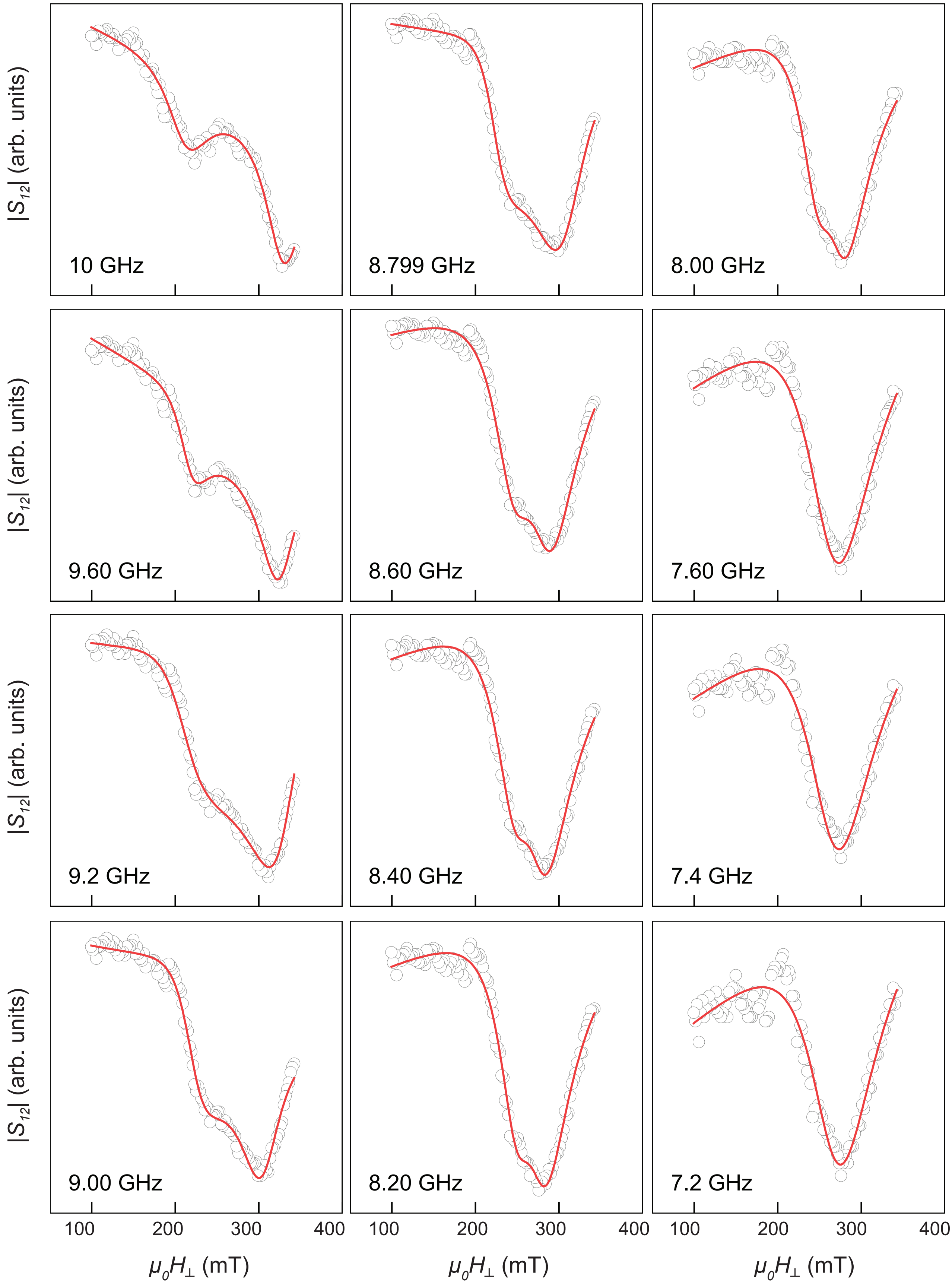

FIG. S3. Field sweep data derived from experimentally obtained frequency sweep data (grey), fitted with a double peak Lorentz function (red) at higher frequencies and a single peak Lorentz function (red) at lower frequencies.

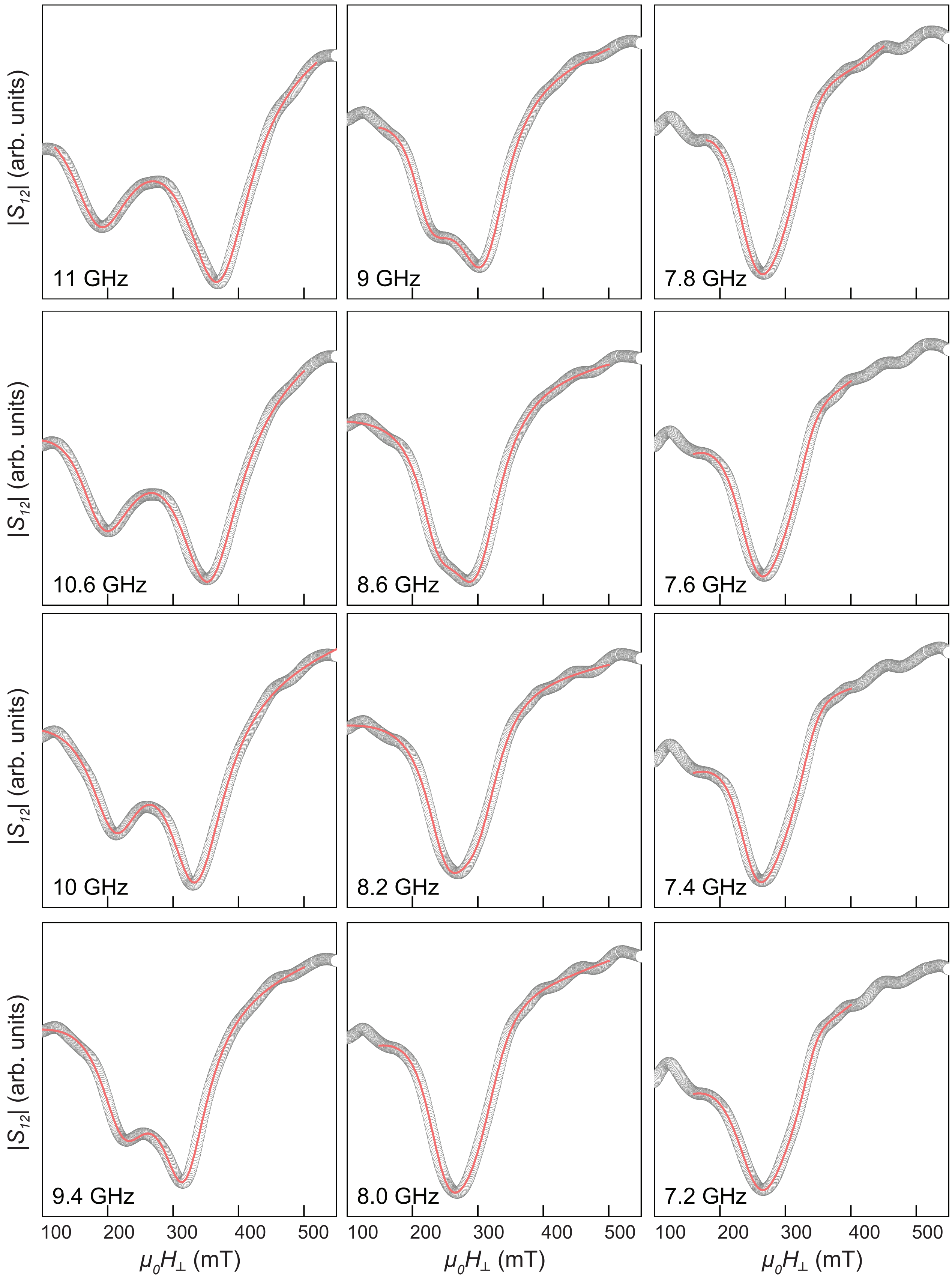

FIG. S4. Experimentally obtained field sweep data (grey) fitted with a double peak Lorentz function (red) at higher frequencies and a single peak Lorentz function (red) at lower frequencies.

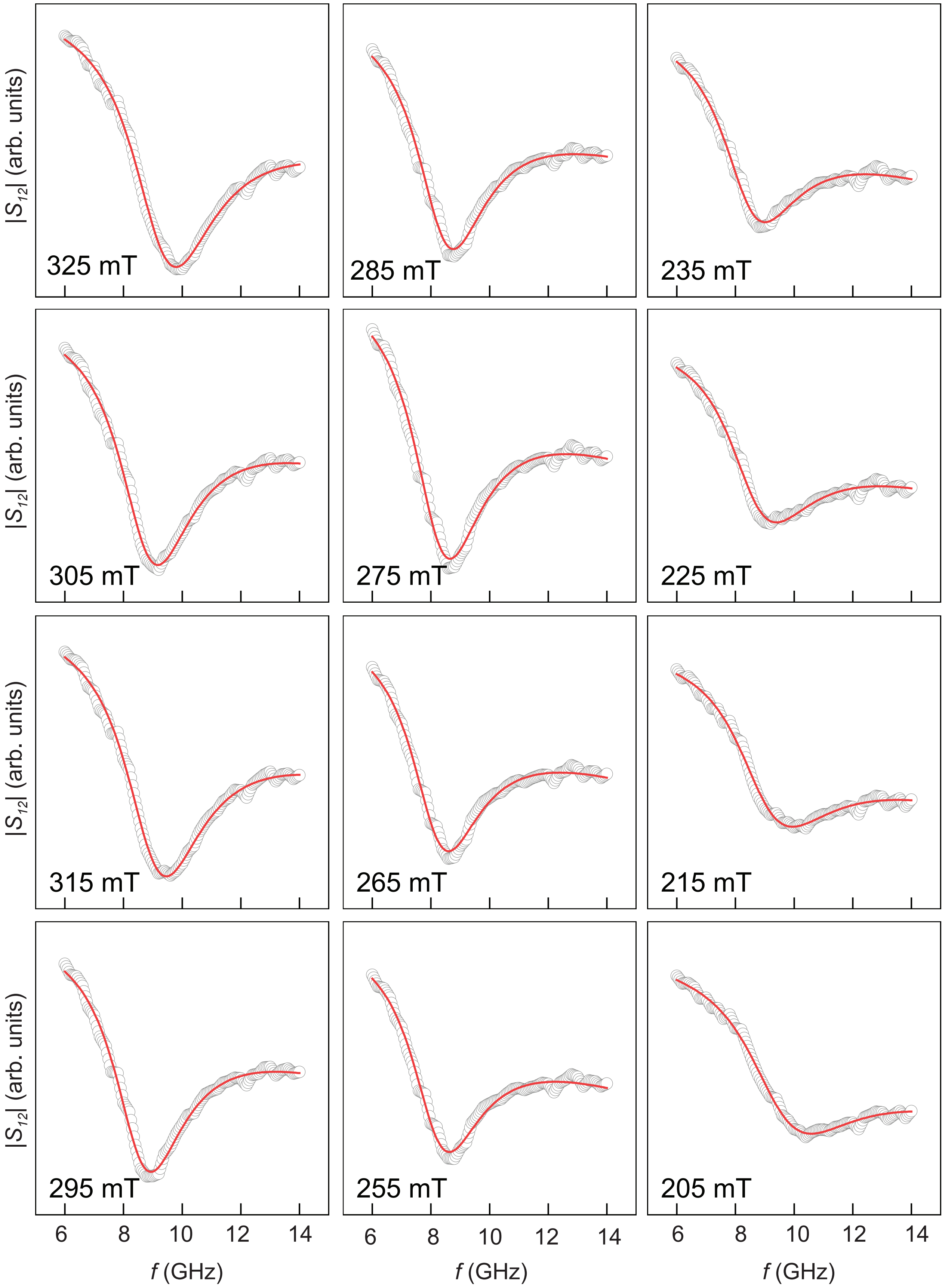

FIG. S5. Frequency sweep data (grey) derived from experimentally obtained field sweep data fitted with a single peak Dysonian function (red).

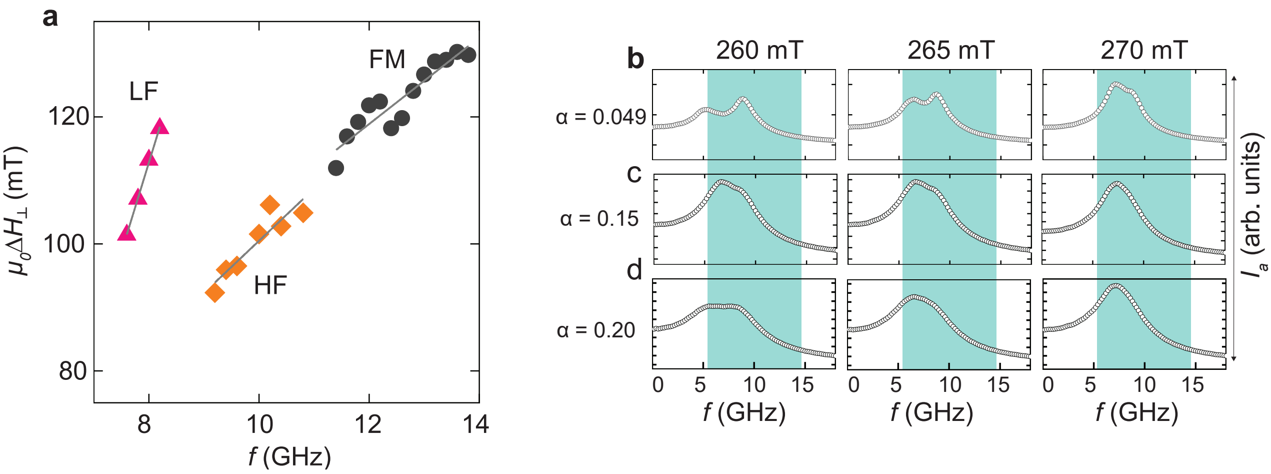

FIG. S6. **a** Frequency dependence of the resonance linewidths for all three experimentally-observed modes, extracted from fits of the raw field sweep data at 300 K. Linear fits yield gradients 28.25, 8.20 and 6.79 mT/GHz for the LF, HF and FM modes, respectively. **b** Micromagnetic simulations of frequency-dependent absorption spectra, performed in the field range where LF and HF modes coexist (260-270 mT) using the same model configuration employed in Fig. 3 of the main text. The simulations use progressively increasing effective dampings $\alpha_{eff} = 0.049$ (obtained from the FM mode in Fig. S1e), 0.15 and 0.20. The shaded region in each spectrum represents the frequency range accessible in our experiments.

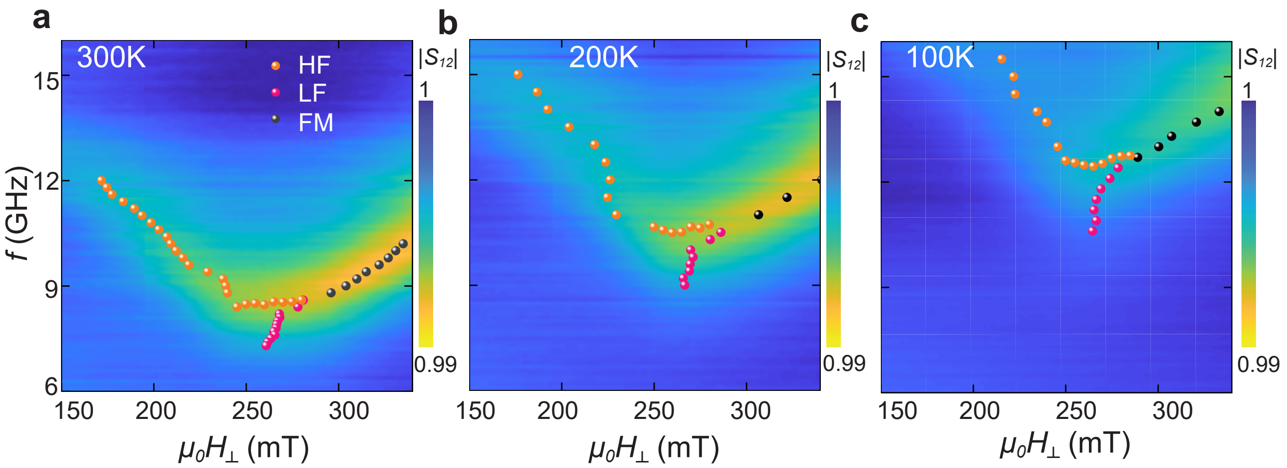

FIG. S7. Temperature dependence of magnetic resonance modes in $[Ir_1Fe_{0.5}Co_{0.5}Pt_1]^{20}$. **a**, **b** and **c** display colour maps of the transmission intensity S_{12} (which is inversely proportional to the microwave absorption) at 300 K, 200 K and 100 K, respectively. The resonance data points are acquired by performing field sweep ferromagnetic resonance experiments at fixed frequencies. The black data points mark the resonance response of field-polarised phase, while the orange and pink data sets indicate the HF and LF resonances in skyrmion phase respectively.

C. Temperature dependence of resonance spectra

Temperature can play an important role in controlling the dynamic properties and resonances of magnetic materials. For example, the magnetic parameters often display a temperature dependence, and thermally-activated magnon modes provide dissipative as well as absorptive channels for microwave excitations. To understand the effects of temperature in $[Ir_1Fe_{0.5}Co_{0.5}Pt_1]^{20}$, we performed field sweep FMR experiments down to 100 K. Variable temperature MFM images [2] have confirmed the presence of skyrmions in these multilayers at low temperatures, although the density of skyrmions decreases with temperature. We duly observe both HF and LF skyrmion resonances at all temperatures down to 100 K, as well as the uniform spin precession in the field-polarised state (Fig. S7). However, the resonance frequencies for all modes shift to higher values as the temperature falls at a rate of $\approx 20\,\mathrm{MHzK^{-1}}$. This shift can be attributed to the temperature dependence of the uniaxial anisotropy K and the saturation magnetisation M_s.

II. MICROMAGNETIC SIMULATIONS

Micromagnetic simulations were performed as described in the main text. Here, we provide further details of our numerical studies.

A. Magnetic parameters for mumax^3 simulations

For the simulations we use the parameters $M_s = 1.02\,\mathrm{MA/m}$, $K = 0.65\,\mathrm{MJ/m^3}$ and $g = 2.17$ (determined experimentally in section I A). The exchange stiffness $A = 9.25\,\mathrm{pJ/m}$ and Dzyaloshinskii-Moriya interaction (DMI) $D = 1.4\,\mathrm{mJ/m^2}$ were determined with the help of an extensive comparison to the experimental resonance spectra as described below. Most simulations were performed with a low Gilbert damping parameter $\alpha = 0.01$ in order to resolve the resonances; some simulations were performed with the experimental value $\alpha = 0.049$. For the determination of the demagnetisation field within mumax^3 in the presence of periodic boundary conditions we use an out-of-plane repetition parameter $N_z = 10$. The in-plane parameters N_x and N_y vary in the range 10-52 depending on the lateral size of the simulated sample. Further details of the simulated geometries and discretisation meshes may be found in the main text and Methods.

B. Determination of exchange stiffness A and Dzyaloshinskii-Moriya interaction D

We performed a large number of simulations on the geometry with a large $2 \times 2\ \mu\mathrm{m}^2$ cross section for various parameters A and D in the range $8\,\mathrm{pJ/m} \leq A \leq 13\,\mathrm{pJ/m}$ and $1.3\,\mathrm{mJ/m^2} \leq D \leq 1.7\,\mathrm{mJ/m^2}$, then compared the obtained absorption spectra to our experimental data. Some examples are shown in Fig. S8, where the symbols correspond to the experimental data points. All these spectra show the characteristic three resonances: FM, HF and LF. As the Kittel resonance frequency is independent of A and D, all spectra coincide at large fields and collapse onto the experimental data. However, at lower fields the Kittel resonance splits into the LF and HF modes attributed to the presence of skyrmions. In the following, we denote the value of the magnetic field where this splitting occurs as the 'splitting field', which increases with D and decreases with A. Note that for large D and small A the frequency of the HF mode does not smoothly connect to the Kittel frequency, but is separated from the other modes by a gap. At the same time the intensity of the HF mode becomes weaker than that of the LF mode. While D has a drastic influence on the shape of the dependencies $f_{\mathrm{LF}}(B)$ and $f_{\mathrm{HF}}(B)$, the exchange constant A mainly influences the relative intensities of the LF and HF modes. For comparison with our experimental data we employ three main criteria: (i) the value of

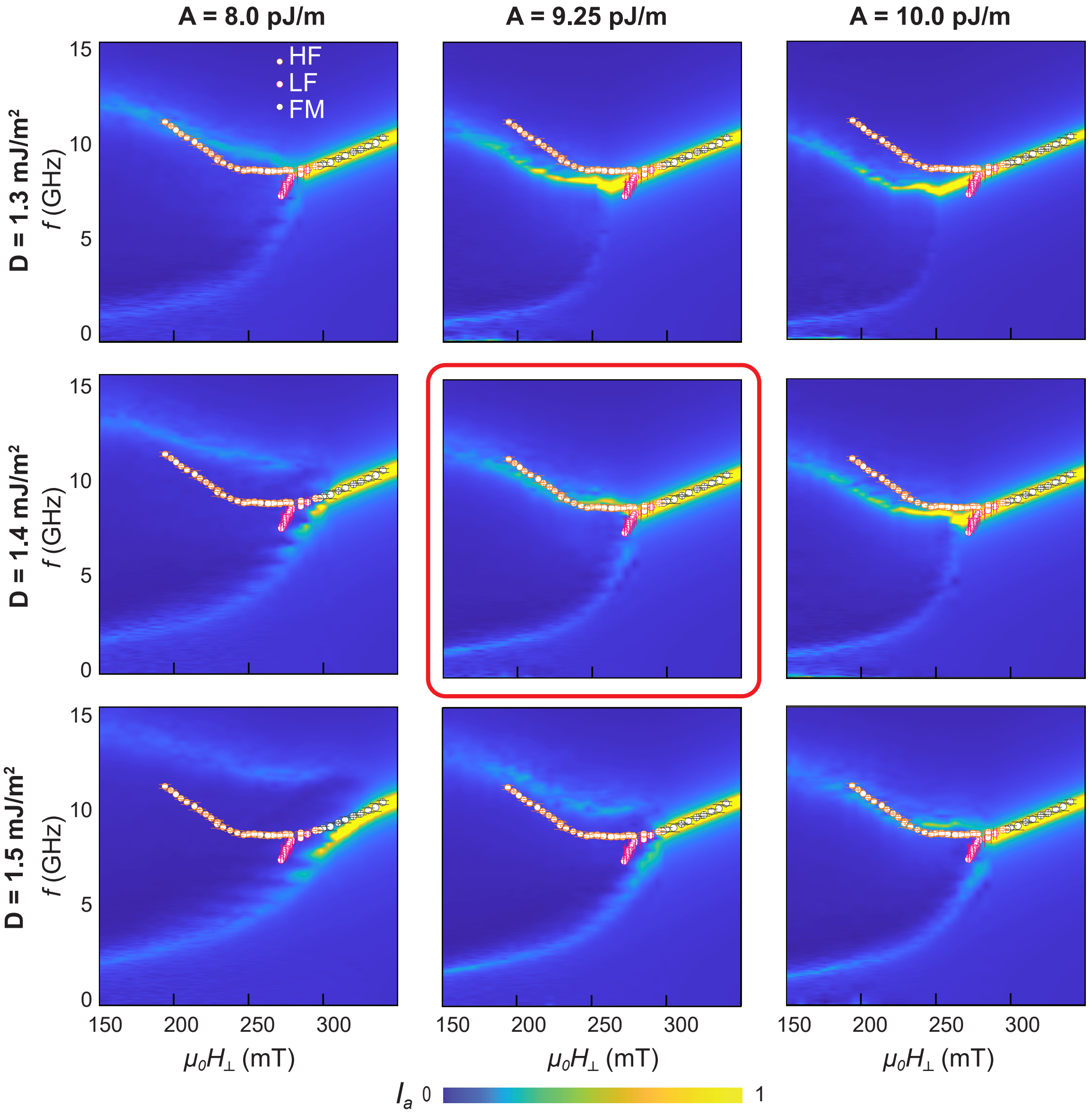

FIG. S8. Simulated resonance spectra in the large 2 × 2 μm^2 geometry using various values for parameters A and D, compared with our experimental data (symbols). All simulations were performed with damping constant $\alpha = 0.01$, uniaxial anisotropy $K = 0.65\,\mathrm{MJ/m^3}$ and saturation magnetisation $M_s = 1.02\,\mathrm{MA/m}$. The choice $A = 9.25\,\mathrm{pJ/m}$, $D = 1.4\,\mathrm{mJ/m^2}$ highlighted in the central panel shows the best agreement.

the splitting field, (ii) the field dependence of the HF resonance frequency $f_{\mathrm{HF}}(B)$ and (iii) the relative intensities of the HF and LF modes. We find an optimal fit for the parameters $A = 9.25\,\mathrm{pJ/m}$ and $D = 1.4\,\mathrm{mJ/m^2}$, corresponding to the central panel in Fig. S8. For these parameters the periodicity of the stripe domains at zero field also agrees well between theory and experiment, as discussed in Fig. 3c and 3d of the main text.

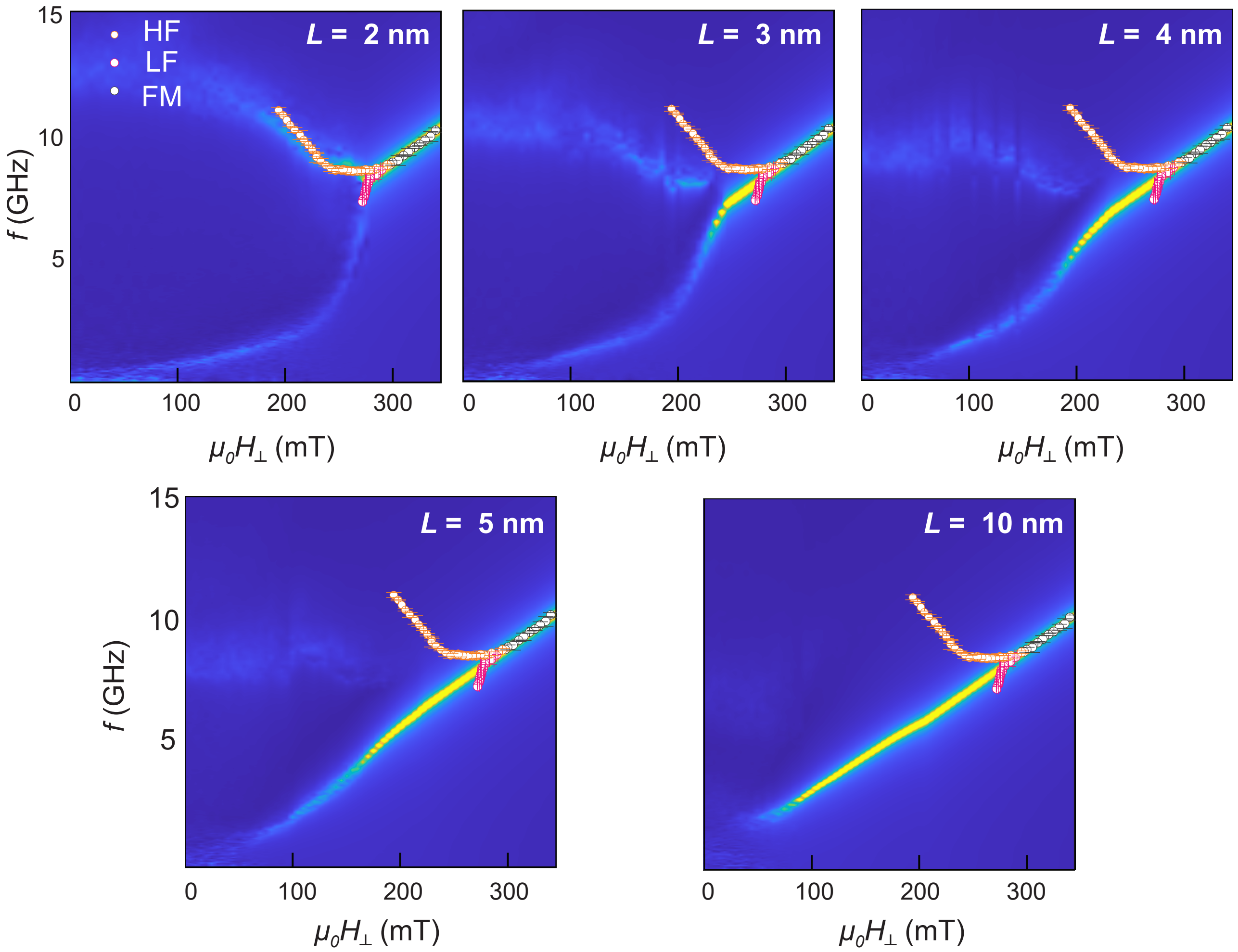

FIG. S9. Simulated resonance spectra in our large $2 \times 2\ \mu\text{m}^2$ geometry model for increasing thicknesses L of the non-magnetic layer. The resonance spectra are distinctly different for small and large L emphasizing the importance of inter-layer dipolar coupling for our experimental setup with $L = 2\,\text{nm}$. In particular, the HF mode is only present for small L.

C. Influence of inter-layer dipolar coupling on the resonance spectra

In order to elucidate the role of inter-layer dipolar coupling we simulated absorption spectra using a model geometry with a $2 \times 2\ \mu\text{m}^2$ cross section and an increasing thickness L of the non-magnetic layer. The results are shown in Fig. S9, where the upper left-panel corresponds to the thickness $L = 2$ nm used in our experimental multilayers. As L increases, the frequency of the HF mode both decreases and loses spectral weight, completely disappearing for large thicknesses. The frequency of the LF mode instead increases with L and also gains intensity. At $L = 10$ nm the frequency of the LF mode almost coincides with the Kittel frequency extrapolated from high fields. Only a small kink indicates the transition between the field-polarised phase at high fields to the skyrmion phase at lower fields. This study agrees with and complements the discussion of Fig. 5 in the main text, where the thickness dependence resonance of the perfectly ordered hexagonal skyrmion lattice has been investigated. The spectra at small

and large thicknesses L are distinctly different, underscoring the central role of the inter-layer coupling whose strength decreases with L.

D. Annealing the metastable disordered skyrmion lattice configuration

As described in detail in the main text, the relaxation from a random initial magnetisation results in a disordered skyrmion lattice configuration even though we simulate a clean system. Examples of such disordered skyrmion configurations are shown in Fig. S10c and Fig. 3b of the main text. These closely resemble the magnetic structures experimentally observed by MFM, although defects and inhomogeneities may also play a role in the experimental system.
At least in the simulations, the disordered skyrmion lattice configuration can be attributed to a complex energy landscape enabling the system to become trapped in a metastable configuration. This metastable configuration can be annealed into a regular hexagonal lattice, as shown in Fig. S10d. The annealing is realised by shaking the system with magnetic field pulses. Two pulses of the form $\mathbf{b}(t) = \mathbf{e}_x b_0 \sin\left(\omega_{max} t^2/t_{sh}\right)$ and $\mathbf{b}(t) = \mathbf{e}_y b_0 \sin\left(\omega_{max} t^2/t_{sh}\right)$ (where the field is pointing along the x- and y-directions respectively) are subsequently applied, each for a time interval $t_{sh} = 10$ ns with $b_0 = 10$ mT and $\omega_{max} = 20$ GHz. Both pulses are repeated four times. Figure S10 compares the resonance spectra of disordered (Fig. S10a) and ordered (Fig. S10b) skyrmion lattices. Importantly, the overall resonance spectrum is quite similar in the two cases, which suggests that the HF and LF modes are indeed characteristic of the perfectly ordered hexagonal lattice.

There are also interesting subtle differences induced by the annealing. First, the Kittel resonance extends to lower fields, and, second, the splitting into the HF and LF modes does not occur smoothly but rather abruptly with clearly visible steps. We recall that the history of the simulation is such that for each field we relax the system from a random magnetisation, which leads to a relaxed metastable state containing a certain number of skyrmions. It is therefore likely that the system is trapped in a wrong topological sector where the number of skyrmions does not correspond to the ground state configuration. When the annealing procedure is applied some of these metastable skyrmions are annihilated above the saturation field, so that the Kittel resonance now extends to lower fields. At the same time the frequency of the LF mode emerges only at lower fields and appears to be separated from the Kittel mode by a gap indicative of a first-order transition - or at least it drops very steeply as a function of decreasing field. The fact that the LF mode is observed experimentally close to the Kittel frequency therefore seems to be attributed to the disordered metastable skyrmion configuration close to the saturation field, as the disorder effectively smoothens out the field dependence of the LF mode

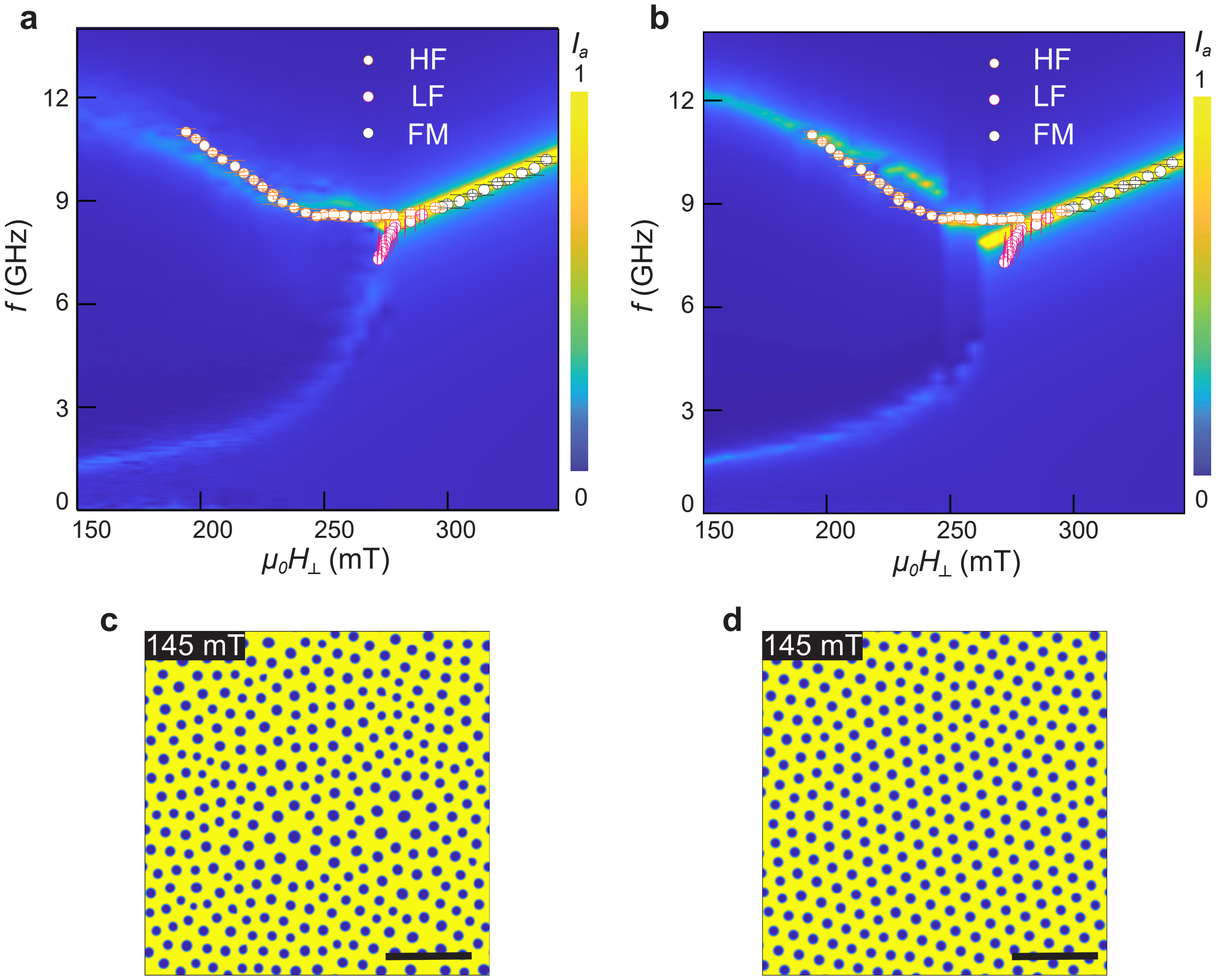

FIG. S10. Annealing the disordered metastable skyrmion configuration into an ordered hexagonal lattice with the help of magnetic field pulses. **a** Resonance spectra obtained for a disordered skyrmion configuration. **b** Resonance spectra of the annealed skyrmion lattice. The Kittel mode now extends to lower fields, and the HF frequency exhibits steps as a function of field close to saturation which are probably due to an incomplete annealing. The intensity in a and b has been normalised for clarity. Overlaid data points are from frequency sweep experiments. **c** and **d** Images of the magnetisation textures at $\mu_0 H_\perp$=145 mT, for the disordered and annealed skyrmion lattices, respectively. The colour code represents the z component of the magnetisation. The scale bar is 500 nm.

frequency. There are additional smaller steps visible in the field dependence of the HF mode frequency in Fig. S10b. We tentatively attribute these latter steps to an incomplete annealing of the system. The annealing procedure is efficient in rearranging skyrmions in real space, but is not optimized to create or annihilate skyrmions. Further studies will be required to elucidate the behaviour close to the thermodynamic transition between the field-polarised state and the skyrmion lattice in this system.

E. Micromagnetic simulations of a metastable state with a single skyrmion in a field-polarised background

In Fig. 4 of the main text we present micromagnetic simulations of a metastable state consisting of a single skyrmion in a field-polarised background. In these simulations the LF mode was observed and its eigenfunction determined (Fig. 4b of the main text). Here, we discuss details of the eigenfunction determination process.

The analysis follows previous work [9] which studied spin waves of a magnetic vortex, and exploits the space-time Fourier transform. The input data obtained from the simulations are (i) the static equilibrium skyrmion solution $\mathbf{m}_0(\mathbf{r})$ and (ii) the time evolution of the excited magnetisation $\mathbf{m}(\mathbf{r}, t)$, where $\mathbf{r}$ is the two-dimensional position vector. First, we determine the first moment of the topological charge density, $\mathbf{R} = \int \mathbf{r}\rho^0_{top}(\mathbf{r})\mathrm{d}\mathbf{r}/\int \rho^0_{top}(\mathbf{r})\mathrm{d}\mathbf{r}$ where $\rho^0_{top}(\mathbf{r}) = \frac{1}{4\pi}\mathbf{m}_0 \cdot [\partial_x \mathbf{m}_0 \times \partial_y \mathbf{m}_0]$, which we identified with the origin of the two-dimensional plane. This allows us to introduce a polar frame of reference (r, χ) centred at $\mathbf{R}$. From the dynamic configuration $\mathbf{m} = \hat{\mathbf{x}} \sin\theta\cos\phi + \hat{\mathbf{y}}\sin\theta\sin\phi + \hat{\mathbf{z}}\cos\theta$ we extract the polar angle $\theta = \theta_0 + \vartheta$ and the azimuthal angle $\phi = \phi_0 + \varphi/\sin\theta_0$, where θ_0 and ϕ_0 are the equilibrium values.

In the linear approximation, the time-dependent functions ϑ and φ parametrise the precession of the magnetisation in the local frame of reference (as depicted in the inset of Fig. 4b in the main text). Since the system is rotationally symmetric the angular momentum μ is a good quantum number, also discussed in section III below. We therefore consider the Fourier transforms

$$\begin{aligned}\hat{F}^{\mu}(r) &= \frac{1}{2\pi T_0}\int_0^{T_0}\mathrm{d}t\int_0^{2\pi}\mathrm{d}\chi\,\vartheta(r,\chi,t)e^{i(\omega_\mu t+\mu\chi)},\\ \hat{G}^{\mu}(r) &= \frac{1}{2\pi T_0}\int_0^{T_0}\mathrm{d}t\int_0^{2\pi}\mathrm{d}\chi\,\varphi(r,\chi,t)e^{i(\omega_\mu t+\mu\chi)},\end{aligned} \tag{3}$$

where T_0 is the total simulation time and ω_μ is the eigenfrequency of the mode with quantum number μ which is known from the evaluated absorption spectrum. The counterclockwise (CCW) LF mode, which is of interest here, has $\mu = -1$.

It can be shown that the Fourier transforms $\hat{F}^{\mu}(r) = \frac{1}{2}f_\mu(r)e^{-i\eta}$ and $\hat{G}^{\mu}(r) = \frac{i}{2}g_\mu(r)e^{-i\eta}$ have a relative phase shift of $\pm\pi/2$ apart from an arbitrary common phase η. The eigenfunctions are characterized by the real functions $f_\mu(r)$ and $g_\mu(r)$ which are displayed for the $\mu = -1$ mode in Fig. 4b of the main text. The function $f_{-1}(r)$ is always positive whereas $g_{-1}(r)$ changes sign

and becomes negative for small distances. The magnitudes of $f_{-1}(r)$ and $g_{-1}(r)$ quantify the semi-major and semi-minor axis of the local precessional ellipse, respectively, and their relative sign determines the orientation of the precession. It is counterclockwise if both functions are positive, and is clockwise when $g_{-1}(r)$ becomes negative. Note however that the precession of the net dipole moment induced by the LF mode is counterclockwise.

F. Micromagnetic simulations of a unit cell of a hexagonal skyrmion lattice

In Fig. S10 it was demonstrated that the HF and LF skyrmion resonances not only occur for the disordered configuration but are also characteristic of the regularly ordered hexagonal lattice. This enabled us to investigate the properties of the resonances in more detail in Fig. 5 of the main text where a rectangular unit cell of the hexagonal lattice containing two skyrmions was simulated. Here we present details of these simulations. We also discuss the relation between the frequency of the LF mode and the skyrmion radius. Moreover, we demonstrate that the system also possesses a breathing mode which can be excited with out-of-plane ac fields.

1. Size of the unit cell and averaged topological density

As explained in the main text, the area of the unit cell $S_{uc} = \sqrt{3}a^2$ with lattice constant a implies an averaged topological density $|\langle\rho_{\text{top}}\rangle| = 2/S_{uc}$. In order to reproduce the magnetic field dependence of the resonances we first determined the averaged topological density from the simulation on the large $2 \times 2\,\mu$m system without annealing. The result is shown in Fig. S11(a) for a spacer thickness $L = 2\,$nm. In the field-polarised state above the saturation field $\sim$ 280 mT the skyrmion density is zero. Upon entering the skyrmion phase at smaller field, the topological density rises sharply and reaches a maximum at $\sim$ 150 mT. Afterwards it decreases approximately linearly with the field. The transition between the skyrmion phase and the stripe labyrinth state cannot be discerned from $\langle\rho_{\text{top}}\rangle$ extracted from the simulations without annealing. However, the field range of the skyrmion phase can be estimated to be 100 mT $< \mu_0 H_\perp <$ 280 mT. The evolution of $\langle\rho_{\text{top}}\rangle$ with the spacer thickness L is shown in panel (b) for various fields. For the simulation of a unit cell, the lattice spacing for a given field value is set such that the resulting topological density coincides with $\langle\rho_{\text{top}}\rangle$ of Fig. S11.

2. Relation between frequency of the LF mode and skyrmion radius R_{sk}

The inset of Fig. 4a in the main text illustrated that the drop in the frequency of the LF mode coincides with an increase of the skyrmion radius R_{sk}. Here we would like to elaborate on the

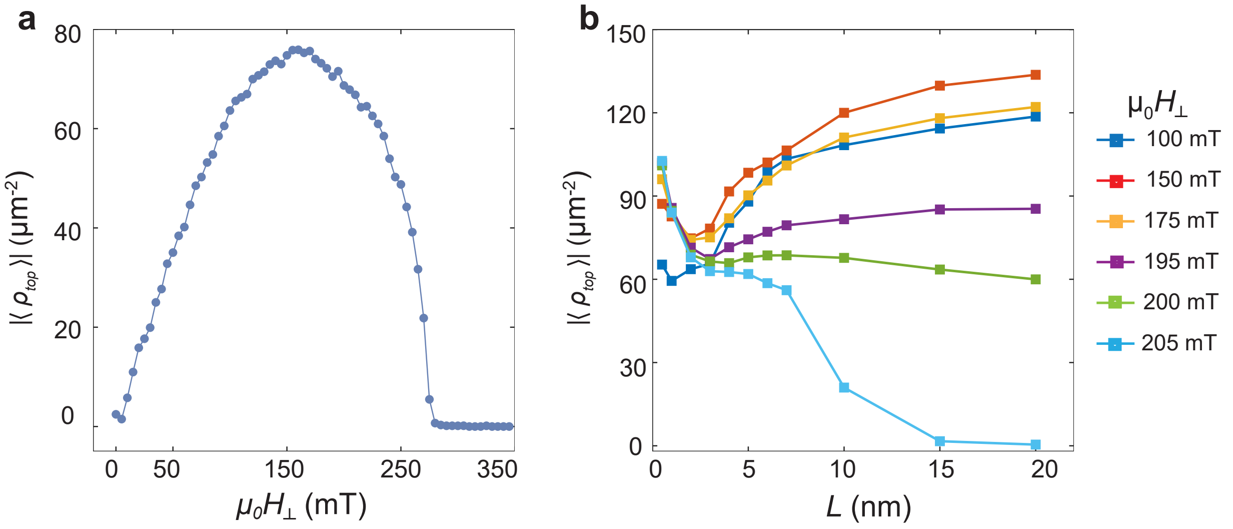

FIG. S11. The average number of skyrmions per unit area $|\langle\rho_{\rm top}\rangle|$ as a function of the applied field $\mu_0 H_\perp$ is shown in panel **a** for the spacer thickness $L = 2\,$nm. Its dependence on the thickness of the non-magnetic spacer is illustrated in panel **b**.

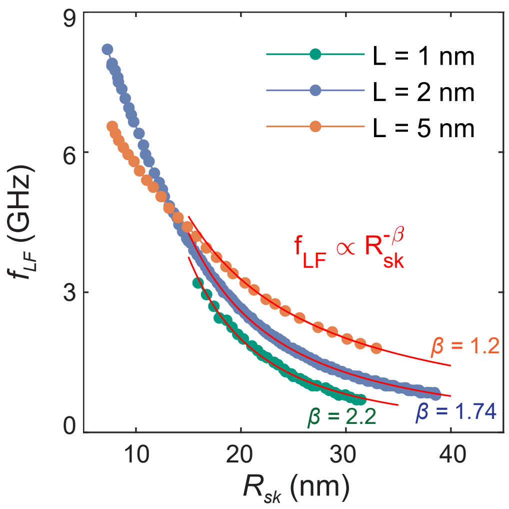

FIG. S12. Relation between the frequency $f_{\rm LF}$ of the LF mode and skyrmion radius R_{sk} obtained from the simulations of a unit cell for different values of spacer thickness L. The frequency decreases with increasing radius and can be fitted for large radii by $f_{\rm LF} \propto R_{sk}^{-\beta}$. The exponent tends to approach $\beta = 1$ for large L, as expected theoretically [10].

connection between these two quantities as obtained from the simulations of the unit cell.

The skyrmion radius can be estimated as follows

$$R_{sk} = \left[\frac{1-\langle m_z\rangle}{2\pi|\langle\rho_{\rm top}\rangle|}\right]^{1/2}. \tag{4}$$

where $\langle m_z\rangle = (S_{uc}M_s)^{-1}\int_{uc} M_z {\rm d}x{\rm d}y$ is the perpendicular magnetisation averaged over the area of the unit cell S_{uc}. The dependence $R_{sk}(B)$ is shown in the inset of Fig. 4a in the main text (denoted by SkL) where it is compared to the skyrmion radius evaluated in a similar manner for

a single skyrmion in a field-polarised background. Remarkably, the latter is always larger than $R_{sk}(B)$ of the skyrmion lattice, implying a substantial effective pressure from the environment.

In Fig. S12 the relation between the frequency of the LF mode and the skyrmion radius is displayed. For large R_{sk}, it can be described by a power-law $f_{\mathrm{LF}} \propto R_{sk}^{-\beta}$ with an exponent β which depends on the thickness L of the non-magnetic spacer. For a single skyrmion in a single layer it was shown in Ref. [10] that the eigenfrequency of the CCW mode possesses an asymptotic behaviour $f_{\mathrm{LF}} \sim R_{sk}^{-1}$ in the limit of large R_{sk}. The trend in Fig. S12 that β approaches 1 for larger L is consistent with this prediction.

3. *Breathing mode in $[Ir_1Fe_{0.5}Co_{0.5}Pt_1]^{20}$ multilayers*

It is well known [11] that the skyrmion lattice also possesses a breathing mode which can be excited by out-of-plane (OP) ac fields. For completeness, we discuss here the breathing mode expected for the present multilayer system.

The numerically determined absorption spectrum for OP ac fields is shown in Fig. S13a. We obtain a breathing mode resonance located in the frequency range ≈ 1-2 GHz. The corresponding magnon probability distribution as defined in the main text is shown in Fig. S13c. It is well localised on a ring close to the skyrmion centre whose radius is on the order of the skyrmion radius (Fig. S13b). A similar magnon probability distribution is obtained for the breathing mode of a single skyrmion (see Ref. [10] as well as Fig. S13c). Video files illustrating the time evolution of all skyrmion modes are included in the Supplementary materials (see section IV).

III. ANALYTICAL THEORY OF A SINGLE SKYRMION IN MULTILAYERS

In order to complement our micromagnetic simulations, in particular those described in section II E and Fig. 4 of the main text, we present here an analytical theory for a single skyrmion in a multilayer stack with an infinite number of magnetic layers of thickness h, separated by non-magnetic layers of thickness L. We discuss the static skyrmion solution in section III B after introducing the model in section III A. Importantly, here we show that the Néel skyrmion is indeed a static solution in the presence of the dipolar interaction in the multilayer. In order to show this, a derivation of the magnetostatic kernel of the multilayer was required, which is presented in Eq. (10). In section III C we derive the magnon spectra, but only in the limit of small magnetic layer thickness $h \to 0$.

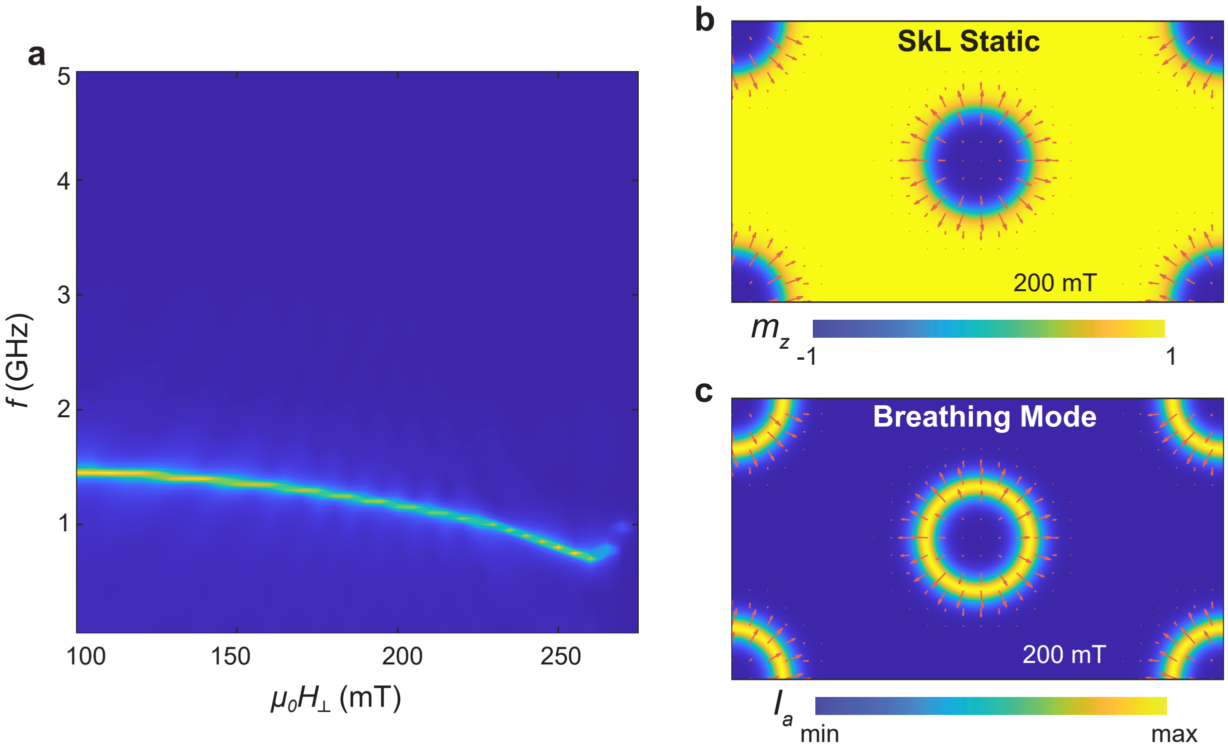

FIG. S13. **a** Absorption spectrum for out-of-plane ac fields simulated using the parameters of our $[Ir_1Fe_{0.5}Co_{0.5}Pt_1]^{20}$ multilayer, predicting a breathing mode in the 1-2 GHz range. **b** Equilibrium configuration of the unit cell at $\mu_0 H_\perp = 200\,\mathrm{mT}$. **c** The magnon probability distribution for the breathing mode is concentrated on a ring around the skyrmion centre.

A. Model

We consider an infinite stack of ferromagnetic layers of thickness h separated by nonmagnetic spacers of thickness L. Thus, the period of the system along the z-axis is $L + h$. For the description of the magnetisation dynamics we neglect damping in this section and limit ourselves to the Landau-Lifshitz equation

$$\partial_t \mathbf{M} = \gamma \left[\mathbf{M} \times \frac{\delta E}{\delta \mathbf{M}} \right], \tag{5}$$

where $\mathbf{M}$ is the magnetisation vector, $\gamma = g\mu_\mathrm{B}/\hbar > 0$ denotes the gyromagnetic ratio, and E is the total energy of the system. We assume that: (i) within each layer the magnetisation is uniform along z and (ii) all layers have the same magnetisation. In the other words, $\mathbf{M}(\mathbf{r}, t) = M_s \mathbf{m}(\boldsymbol{\varrho}, t) S(z)$, where M_s is the saturation magnetisation, $\mathbf{m}(\boldsymbol{\varrho}, t)$ is the unit magnetisation vector within a layer with $\boldsymbol{\varrho} = x\hat{\mathbf{x}} + y\hat{\mathbf{y}}$ being the position vector within the xy-plane, $S(z) = 1$ for $z \in$ [ferromagnet] and $S(z) = 0$ for $z \in$ [spacer]. In this case, the total energy of the system

simply reads $E = NE_1$ where $N \to \infty$ is the number of the layers and

$$E_1 = h \int \left[A \sum_{i=x,y,z} (\boldsymbol{\nabla} m^i)^2 - K(m^z)^2 - \mu_0 H M_s m^z + D\mathcal{E}_{\text{DMI}} - \frac{\mu_0 M_s}{2h} \int\limits_0^h \mathbf{m} \cdot \mathbf{H}_m \mathrm{d}z \right] \mathrm{d}\boldsymbol{\varrho} \tag{6}$$

is the energy of a single ferromagnetic layer. Here, we include the following contributions to the total energy: isotropic exchange with stiffness A, uniaxial anisotropy with constant K, external magnetic field $\mathbf{H} = H\hat{\mathbf{z}}$, Dzyaloshinskii-Moriya interaction (DMI) of the interfacial type $\mathcal{E}_{\text{DMI}} = m^z \boldsymbol{\nabla} \cdot \mathbf{m} - \mathbf{m} \cdot \boldsymbol{\nabla} m^z$ with constant D, and interaction with the self-induced magnetostatic field

$$\mathbf{H}_m(\mathbf{r}) = -\frac{M_s}{4\pi} \boldsymbol{\nabla} \int (\mathbf{m}(\boldsymbol{r}') \cdot \boldsymbol{\nabla}') \frac{\mathrm{d}\mathbf{r}'}{|\mathbf{r} - \mathbf{r}'|}, \tag{7}$$

where the integration is taken over the total magnetic volume, i.e. over all the layers.

B. Static skyrmion solution

Here, we consider the static skyrmion solution in the background of the uniformly polarised state. One can show that the static limit of equation (5) possesses the Néel skyrmion as an equilibrium solution. Introducing the angular parametrisation $\mathbf{m} = \sin\theta\cos\phi\hat{\mathbf{x}} + \sin\theta\sin\phi\hat{\mathbf{y}} + \cos\theta\hat{\mathbf{z}}$ the equations $\mathbf{M} \times \frac{\delta E}{\delta \mathbf{M}} = 0$ are simultaneously fulfilled for $\theta = \Theta(\rho)$ and $\phi = \chi$ for $D > 0$, and $\phi = \chi + \pi$ for $D < 0$, where $\Theta(\rho)$ is determined by the following integro-differential equation

$$\begin{aligned} &\Delta_\rho \Theta - \sin\Theta\cos\Theta \left(\frac{1}{\rho^2} + Q \right) - b\sin\Theta + \frac{|d|}{\rho} \sin^2\Theta \\ &+ \frac{1}{2} \int\limits_0^\infty \rho' \left[G_{\rho\rho}(\rho, \rho') \cos\Theta(\rho) \sin\Theta(\rho') - G_{zz}(\rho, \rho') \sin\Theta(\rho) \cos\Theta(\rho') \right] \mathrm{d}\rho' = 0, \\ &\qquad \Theta(0) = \pi, \qquad \Theta(\infty) = 0. \end{aligned} \tag{8}$$

Here $\{\rho, \chi\}$ is the polar frame of reference with $\rho = r/\ell$ being the dimensionless radial distance to the skyrmion centre, where $\ell = \sqrt{A/(\mu_0 M_s^2)}$ is the exchange length. $b = H/H_0$ is the normalized magnetic field with $\mu_0 H_0 = 2\mu_0 M_s$, and the quality factor $Q = K/(\mu_0 M_s^2)$ represents the anisotropy strength with respect to the magnetostatic effect. $\Delta_\rho f = \rho^{-1}\partial_\rho(\rho\partial_\rho f)$ is the radial part of the Laplacian, and $d = D/\sqrt{\mu_0 M_s^2 A}$ is the dimensionless DMI constant.

The fact that the Néel-type skyrmion is an exact solution of the static Landau-Lifshitz equations in the presence of the dipolar interaction is a non-trivial result. While the DMI favours Néel-type spin twisting, the magnetostatic interaction favours instead a Bloch-type arrangement.

As a result, a skyrmion with a helicity intermediate between Néel- and Bloch-type could have been expected. At the moment we cannot exclude the existence of such an additional solution analytically. However, our numerical simulations confirmed the Néel skyrmion configuration, and did not yield additional solutions. The integral in Eq. (8) represents the contribution from the dipole-dipole interaction. The Green functions G_{zz} and $G_{\rho\rho}$ correspond to the contributions of the surface and volume magnetostatic charges, respectively. They have the following form

$$
\begin{aligned}
G_{zz}(\rho,\rho') &= \int_0^\infty k\left[1-kh\mathfrak{g}(kh,kL)\right]J_0(\rho k)J_0(\rho' k)\mathrm{d}k,\\
G_{\rho\rho}(\rho,\rho') &= h\int_0^\infty k^2\mathfrak{g}(kh,kL)J_1(\rho k)J_1(\rho' k)\mathrm{d}k
\end{aligned}
\tag{9}
$$

with $J_\nu(x)$ being the Bessel functions of the first kind. The surface part G_{zz} appears in many problems involving magnetic vortices [12–15] and Bloch skyrmions [16] in ferromagnetic films. The additional volume contribution $G_{\rho\rho}$ is attributed to the volume magnetic charges generated by the Néel skyrmion [16]. The magnetostatic kernel

$$
\mathfrak{g}(\xi,\eta) = \frac{e^{-\xi}-1+\xi}{\xi^2} + \frac{\sinh^2(\xi/2)}{(\xi/2)^2}\frac{1}{e^{\xi+\eta}-1}, \tag{10}
$$

is determined solely by the geometry of the multilayer. In the limiting case $L\to\infty$, which corresponds to a single layer, the kernel (10) reduces to the form $\mathfrak{g}(\xi,\infty) = (e^{-\xi}-1+\xi)\xi^{-2}$ which was previously obtained in Ref. [17] for the problem of spin waves in ferromagnetic plates. In the opposite limit of vanishing non-magnetic spacers, $L=0$, where the system reduces to a bulk magnet, the kernel (10) is reduced to $\mathfrak{g}(\xi,0) = 1/\xi$. This coincides with the Green function of the magnetostatic potential for a 3D ferromagnet. Eq. (8) determines the skyrmion profile and requires a numerical analysis which is technically rather complicated due to the nonlocality of the problem. In order to make progress we therefore consider the limit $h\to 0$ in the following. In this case the problem becomes local and independent of the precise value of $L>0$, i.e. the thickness of the non-magnetic spacer:

$$
\lim_{h\to 0} G_{\rho\rho}(\rho,\rho') = 0, \qquad \lim_{h\to 0} G_{zz}(\rho,\rho') = \frac{\delta(\rho-\rho')}{\rho}, \tag{11}
$$

where $\delta(x)$ is the Dirac delta-function. In this limit Eq. (8) reduces to the well known [18, 19]

equation for the skyrmion profile

$$\begin{aligned}&\Delta_\rho\Theta - \sin\Theta\cos\Theta\left(\frac{1}{\rho^2}+\tilde{Q}\right) - b\sin\Theta + \frac{|d|}{\rho}\sin^2\Theta = 0\\&\Theta(0)=\pi, \qquad \Theta(\infty)=0.\end{aligned} \tag{12}$$

where $\tilde{Q} = Q - \frac{1}{2}$. This anisotropy shift originates from the local part of the magnetostatic interaction. The latter is represented by the non-vanishing term G_{zz} in Eq. (11), and can be understood as the energy of an infinitely thin capacitor, where the role of electrical charges is mimicked by the surface magnetostatic charges on the top and bottom surfaces of the magnetic layer.

The orange line with dots in Fig. S14(a) shows the skyrmion profile $m_z(\rho)$ as obtained from the numerical simulations for the applied magnetic field $\mu_0 H_\perp = 250\,\mathrm{mT}$, see also the discussion in Fig. 4 of the main text. The dashed line is the corresponding result obtained analytically in the limit of $h \to 0$ where the dipolar interaction becomes local, see Eqs. (11). Note that in this limit the theory becomes independent of L, i.e., the thickness of the non-magnetic spacer. The two results drastically differ, in particular, the skyrmion radius is approximately a factor of four smaller without the non-local dipolar interaction. This again underscores the importance of inter-layer dipolar interactions in the experimental system.

A skyrmion profile with a radius similar to the numerical simulations is obtained analytically in the limit $h \to 0$ by changing the uniaxial anisotropy to the value $K = 0.32\ \mathrm{MJ/m^3}$, setting the magnetic field H to zero, and maintaining the values of all other parameters. The result is shown in Fig. S14(a) by the green solid line.

C. Spin wave excitation in the presence of a skyrmion

In this section we consider the linear spin wave excitations in the presence of the skyrmion. We again limit ourselves to the limit $h \to 0$, i.e. a vanishing magnetic layer thickness. The solution of this problem is well-known from the literature [10, 20] and closely follows previous works on spin wave excitations in the presence of vortices [21–24]. We sketch here only the main steps.

We introduce small deviations to the static state $\theta(\rho,\chi,\tau) = \Theta(\rho) + \vartheta(\rho,\chi,\tau)$ and $\phi(\rho,\chi,\tau) = \chi + \varphi(\rho,\chi,\tau)/\sin\Theta(\rho)$ and linearise the equation of motion (5) with respect to ϑ and φ. One can show [10] that the solutions possesses the form $\vartheta(\rho,\chi,\tau) = f_\mu(\rho)\cos(\omega\tau + \mu\chi + \eta)$ and $\varphi(\rho,\chi,\tau) = g_\mu(\rho)\sin(\omega\tau + \mu\chi + \eta)$, where $\mu \in \mathbb{Z}$ is the angular quantum number and η is an arbitrary phase shift. For a given μ the eigenfunctions f_μ and g_μ are determined by the

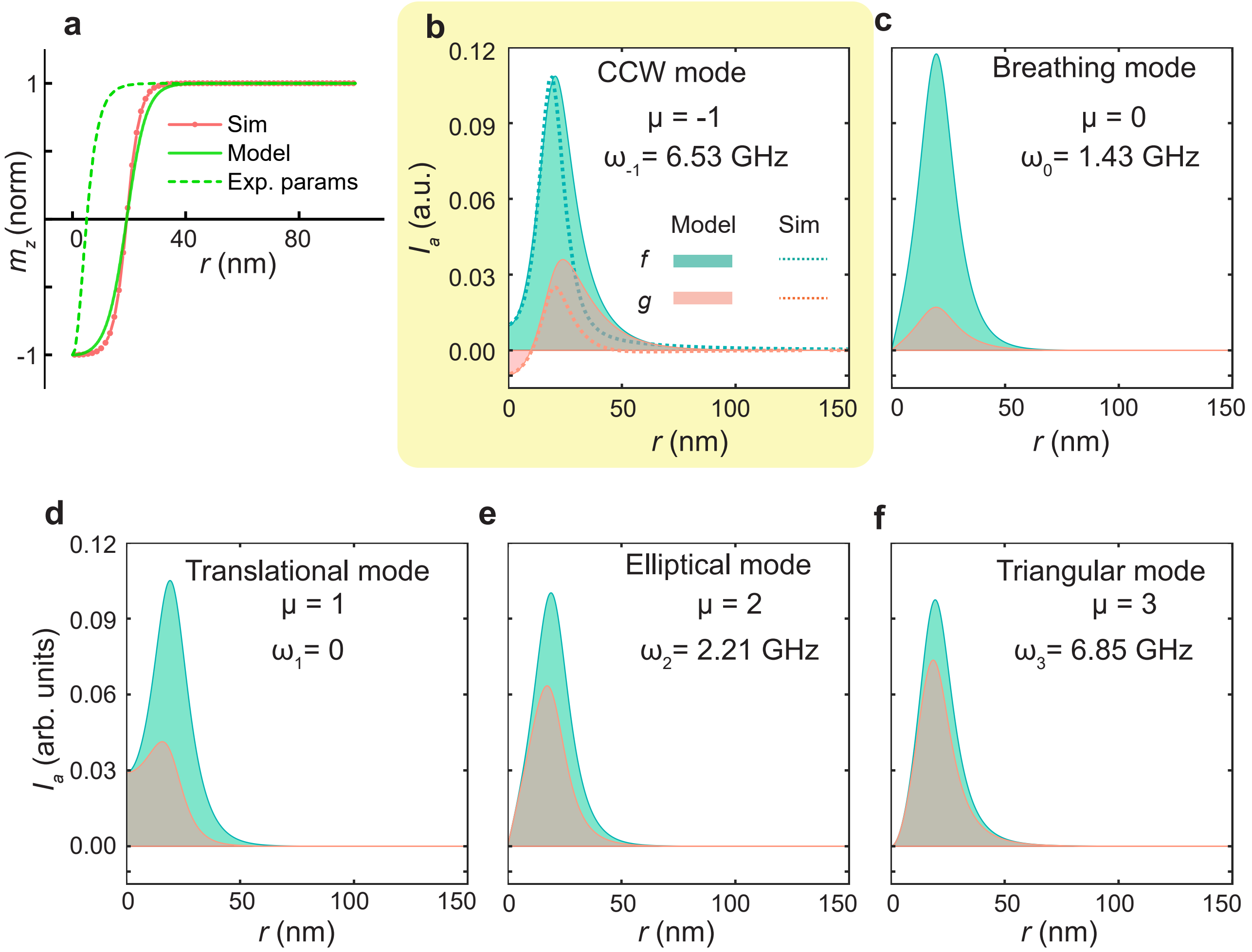

FIG. S14. **a** Magnetisation profiles of a single skyrmion in equilibrium for the multilayer system. The orange line is obtained by means of micromagnetic simulations with $\mu_0 H_\perp = 250\,\mathrm{mT}$, see section II E. The dashed green line corresponds to the analytical result for the same parameters, but in the limit of magnetic spacer thickness $h \to 0$. The drastic difference is attributed to the inter-layer coupling. The solid line is an analytical solution but for a modified uniaxial anisotropy $K = 0.32\ \mathrm{MJ/m^3}$ and zero field $H = 0$. **b-f** Eigenfunctions of all localised eigenstates of the latter skyrmion (solid green line in **a**). Only two of these modes have nonzero net magnetic moment: the counterclockwise (CCW) mode $\mu = -1$ and the breathing mode $\mu = 0$, where the moment is oscillating in-plane and out-of-plane, respectively.

following eigenvalue problem

$$\hat{\mathrm{H}}_\mu \boldsymbol{\psi}_\mu = \omega \hat{\sigma}_x \boldsymbol{\psi}_\mu. \tag{13}$$

Here $\boldsymbol{\psi}_\mu = (f_\mu, g_\mu)^{\mathrm{T}}$ and the Hamiltonian is given by

$$\hat{\mathrm{H}}_\mu = \begin{pmatrix} -\Delta_\rho + \frac{\mu^2}{\rho^2} + U_1 & \mu W \\ \mu W & -\Delta_\rho + \frac{\mu^2}{\rho^2} + U_2 \end{pmatrix}. \tag{14}$$

Here, $\hat{\sigma}_x$ is the first Pauli matrix and the potentials U_1, U_2 and W read

$$
\begin{aligned}
U_1 &= \cos 2\Theta \left(\frac{1}{\rho^2} + \tilde{Q}\right) + b\cos\Theta - \frac{d}{\rho}\sin 2\Theta, \\
U_2 &= \cos^2\Theta \left(\frac{1}{\rho^2} + \tilde{Q}\right) + b\cos\Theta - (\partial_\rho\Theta)^2 - d\left(\partial_\rho\Theta + \frac{\sin\Theta\cos\Theta}{\rho}\right), \\
W &= 2\frac{\cos\Theta}{\rho^2} - d\frac{\sin\Theta}{\rho}.
\end{aligned}
\tag{15}
$$

The radial profile of the mode is determined by the eigenfunctions f_μ and g_μ. The solutions for the skyrmion with modified uniaxial anisotropy at zero field corresponding to the solid green line in Fig. S14(a) are shown in panels (b)-(f). Five eigenstates with localised eigenfunctions are found. Only the CCW mode with $\mu = -1$ is susceptible to an in-plane uniform ac magnetic field whereas the breathing mode can be excited by an out-of-plane ac field. The translational mode with $\mu = 1$ has zero frequency. The other modes with $\mu = 2, 3$ are associated with higher order multipoles and cannot be excited in a FMR experiment. The wavefunction of the CCW mode with $\mu = -1$ is very close to the profiles extracted numerically, shown by the dashed lines in Fig. S14(b) (see also Fig. 4b in the main text). Based on this and also on the fact that the LF mode has a CCW rotating magnetic moment, we conclude that the LF mode is akin to the $\mu = -1$ CCW mode of a single skyrmion.

IV. SUPPLEMENTARY VIDEOS

We attach videos obtained from the micromagnetic simulations of configuration 1 (a metastable state containing a single skyrmion in a field-polarised background) and configuration 2 (corresponding to the unit cell of a hexagonal skyrmion lattice containing two skyrmions).

A. LF oscillations for configuration 1: a single skyrmion in a field-polarised background

1. IsolatedSk.gif: CCW oscillations of an isolated skyrmion exhibiting LF resonance with an oscillation period of 345 ps.

B. HF, LF and breathing (BR) oscillations for configuration 2: a unit cell of a hexagonal skyrmion lattice

Following videos display oscillations in the IP and OP magnetisation of a unit cell containing two skyrmions during HF, LF and BR mode resonances. The unit cell was relaxed at 200 mT

prior to applying an ac magnetic field.

1. UCHFMx.gif: Oscillations of the IP magnetisation m_x at 10.6 GHz excited by an IP ac magnetic field.

2. UCHFMz.gif: Oscillations of the OP magnetisation m_z at 10.6 GHz excited by an IP ac magnetic field.

3. UCLFMx.gif: Oscillations of the IP magnetisation m_x at 2 GHz excited by an IP ac magnetic field.

4. UCLFMz.gif: Oscillations of the OP magnetisation m_z at 2 GHz excited by an IP ac magnetic field.

5. UBRFMz.gif: Dynamics of the breathing mode at 1.15 GHz excited by an OP ac magnetic field.

---

[1] A. Soumyanarayanan, M. Raju, A. L. Gonzalez Oyarce, A. K. C. Tan, M.-Y. Im, A. P. Petrović, P. Ho, K. H. Khoo, M. Tran, C. K. Gan, F. Ernult, and C. Panagopoulos, Tunable room-temperature magnetic skyrmions in Ir/Fe/Co/Pt multilayers, Nature Materials **16**, 898 (2017).

[2] M. Raju, A. Yagil, A. Soumyanarayanan, A. K. C. Tan, A. Almoalem, F. Ma, O. M. Auslaender, and C. Panagopoulos, The evolution of skyrmions in Ir/Fe/Co/Pt multilayers and their topological Hall signature, Nature Communications **10**, 696 (2019).

[3] N. K. Duong, M. Raju, A. P. Petrović, R. Tomasello, G. Finocchio, and C. Panagopoulos, Stabilizing zero-field skyrmions in Ir/Fe/Co/Pt thin film multilayers by magnetic history control, Applied Physics Letters **114**, 072401 (2019).

[4] D. Ehlers, I. Stasinopoulos, V. Tsurkan, H.-A. Krug von Nidda, T. Fehér, A. Leonov, I. Kézsmárki, D. Grundler, and A. Loidl, Skyrmion dynamics under uniaxial anisotropy, Physical Review B **94**, 014406 (2016).

[5] N. Mecking, Y. S. Gui, and C.-M. Hu, Microwave photovoltage and photoresistance effects in ferromagnetic microstrips, Physical Review B **76**, 224430 (2007).

[6] M. Harder, Z. X. Cao, Y. S. Gui, X. L. Fan, and C.-M. Hu, Analysis of the line shape of electrically detected ferromagnetic resonance, Physical Review B **84**, 054423 (2011).

[7] H. T. Nembach, T. J. Silva, J. M. Shaw, M. L. Schneider, M. J. Carey, S. Maat, and J. R.

Childress, Perpendicular ferromagnetic resonance measurements of damping and Landé g-factor in sputtered $(Co_2Mn)_{1\text{-x}}Ge_x$ films, Physical Review B **84**, 054424 (2011).

[8] L. Rózsa, J. Hagemeister, E. Y. Vedmedenko, and R. Wiesendanger, Effective damping enhancement in noncollinear spin structures, Physical Review B **98**, 100404 (2018).

[9] M. Buess, R. Höllinger, T. Haug, K. Perzlmaier, U. Krey, D. Pescia, M. R. Scheinfein, D. Weiss, and C. H. Back, Fourier Transform Imaging of Spin Vortex Eigenmodes, Physical Review Letters **93**, 077207 (2004).

[10] V. P. Kravchuk, D. D. Sheka, U. K. Rößler, J. van den Brink, and Y. Gaididei, Spin eigenmodes of magnetic skyrmions and the problem of the effective skyrmion mass, Physical Review B **97**, 064403 (2018).

[11] M. Garst, J. Waizner, and D. Grundler, Collective spin excitations of helices and magnetic skyrmions: review and perspectives of magnonics in non-centrosymmetric magnets, Journal of Physics D: Applied Physics **50**, 293002 (2017).

[12] E. Feldtkeller and H. Thomas, *Phys. kondens. Materie*, Tech. Rep. (1965).

[13] K. Y. Guslienko, B. A. Ivanov, V. Novosad, Y. Otani, H. Shima, and K. Fukamichi, Eigenfrequencies of vortex state excitations in magnetic submicron-size disks, Journal of Applied Physics **91**, 8037 (2002).

[14] K. Y. Guslienko and V. Novosad, Vortex state stability in soft magnetic cylindrical nanodots, Journal of Applied Physics **96**, 4451 (2004).

[15] Y. Gaididei, V. P. Kravchuk, and D. D. Sheka, Magnetic vortex dynamics induced by an electrical current, International Journal of Quantum Chemistry **110**, 83 (2010).

[16] K. Guslienko and Z. Gareeva, Magnetic skyrmion low frequency dynamics in thin circular dots, Journal of Magnetism and Magnetic Materials **442**, 176 (2017).

[17] K. Y. Guslienko and A. N. Slavin, Spin-waves in cylindrical magnetic dot arrays with in-plane magnetization, Journal of Applied Physics **87**, 6337 (2000).

[18] A. O. Leonov, T. L. Monchesky, N. Romming, A. Kubetzka, A. N. Bogdanov, and R. Wiesendanger, The properties of isolated chiral skyrmions in thin magnetic films, New Journal of Physics **18**, 065003 (2016).

[19] A. Bogdanov and A. Hubert, Thermodynamically stable magnetic vortex states in magnetic crystals, Journal of Magnetism and Magnetic Materials **138**, 255 (1994).

[20] C. Schütte and M. Garst, Magnon-skyrmion scattering in chiral magnets, Physical Review B **90**, 094423 (2014).

[21] D. D. Sheka, B. A. Ivanov, and F. G. Mertens, Internal modes and magnon scattering on topological solitons in two-dimensional easy-axis ferromagnets, Physical Review B **64**, 024432 (2001).

[22] B. A. Ivanov and D. D. Sheka, Local magnon modes and the dynamics of a small-radius two-dimensional magnetic soliton in an easy-axis ferromagnet, Journal of Experimental and Theoretical Physics Letters **82**, 436 (2005).

[23] B. A. Ivanov, H. J. Schnitzer, F. G. Mertens, and G. M. Wysin, Magnon modes and magnon-vortex scattering in two-dimensional easy-plane ferromagnets, Physical Review B **58**, 8464 (1998).

[24] D. D. Sheka, I. A. Yastremsky, B. A. Ivanov, G. M. Wysin, and F. G. Mertens, Amplitudes for magnon scattering by vortices in two-dimensional weakly easy-plane ferromagnets, Physical Review B **69**, 054429 (2004).
